# Healthcare provider perspectives on delivering next generation rotavirus vaccines in five low-to-middle-income countries

**DOI:** 10.1371/journal.pone.0270369

**Published:** 2022-06-23

**Authors:** Jessica Mooney, Jessica Price, Carolyn Bain, John Tanko Bawa, Nikki Gurley, Amresh Kumar, Guwani Liyanage, Rouden Esau Mkisi, Chris Odero, Karim Seck, Evan Simpson, William P. Hausdorff

**Affiliations:** 1 PATH, Seattle, Washington, United States of America; 2 PATH, Accra, Ghana; 3 PATH, New Delhi, India; 4 Department of Pediatrics, Faculty of Medical Sciences, University of Sri Jayewardenepura, Nugegoda, Sri Lanka; 5 PATH, Lilongwe, Malawi; 6 PATH, Nairobi, Kenya; 7 Independent Consultant, Dakar, Sénégal; 8 PATH, Washington, D.C., United States of America, and Université Libre de Bruxelles, Brussels, Belgium; The Technical University of Kenya, KENYA

## Abstract

**Background:**

Live oral rotavirus vaccines (LORVs) have significantly reduced rotavirus hospitalizations and deaths worldwide. However, LORVs are less effective in low- and middle-income countries (LMICs). Next-generation rotavirus vaccines (NGRVs) may be more effective but require administration by injection or a neonatal oral dose, adding operational complexity. Healthcare providers (HPs) were interviewed to assess rotavirus vaccine preferences and identify delivery issues as part of an NGRV value proposition.

**Objective:**

Determine HP vaccine preferences about delivering LORVs compared to injectable (iNGRV) and neonatal oral (oNGRV) NGRVs.

**Methods:**

64 HPs from Ghana, Kenya, Malawi, Peru, and Senegal were interviewed following a mixed-method guide centered on three vaccine comparisons: LORV vs. iNGRV; LORV vs. oNGRV; oNGRV vs. iNGRV. HPs reviewed attributes for each vaccine in the comparisons, then indicated and explained their preference. Additional questions elicited views about co-administering iNGRV+LORV for greater public health impact, a possible iNGRV-DTP-containing combination vaccine, and delivering neonatal doses.

**Results:**

Almost all HPs preferred oral vaccine options over iNGRV, with many emphasizing an aversion to additional injections. Despite this strong preference, HPs described challenges delivering oral doses. Preferences for LORV vs. oNGRV were split, marked by disparate views on rotavirus disease epidemiology and the safety, need, and feasibility of delivering neonatal vaccines. Although overwhelmingly enthusiastic about an iNGRV-DTP-containing combination option, several HPs had concerns. HP views were divided on the feasibility of co-administering iNGRV+LORV, citing challenges around logistics and caregiver sensitization.

**Conclusion:**

Our findings provide valuable insights on delivering NGRVs in routine immunization. Despite opposition to injectables, openness to co-administering LORV+iNGRV to improve efficacy suggests future HP support of iNGRV if adequately informed of its advantages. Rationales for LORV vs. oNGRV underscore needs for training on rotavirus epidemiology and stronger service integration. Expressed challenges delivering existing LORVs merit further examination and indicate need for improved delivery.

## Introduction

More than 100 countries worldwide have introduced live oral rotavirus vaccines (LORVs) in national immunization programs to-date [1]. LORVs have significantly reduced diarrheal disease morbidity and mortality [2], though their protection against severe disease in low-and middle-income countries (LMICs) is substantially lower (50–60%) compared to high-income countries (80–95%) [3–10]. Furthermore, evidence suggests declining immunity after the first year of vaccination. Clinical trials in Kenya, Mali, Malawi, Ghana, South Africa, and Bangladesh suggest a 30%–40% decline from the first to second year of life [5–15]. Though some degree of lower effectiveness in low-income settings may be attributed to undocumented high incidence of rotavirus circulating among the population, these observations, while important, do not sufficiently explain the marked difference in LORV effectiveness between high- and low-income countries [16]. Additionally, LORVs have been associated with a slightly elevated risk of intussusception two to seven days after vaccination in some settings [17].

Next-generation rotavirus vaccines (NGRVs) in the vaccine development pipeline, including neonatal oral candidates and injectable candidates [18–20], may help address the disparity in LORV effectiveness and have the potential to mitigate or eliminate the risk of intussusception. However, attributes of different NGRVs have programmatic implications, including their acceptability to caregivers and providers as well as the feasibility to deliver them within existing immunization schedules. Healthcare providers are well positioned to provide unique, on-the-ground insights for how best to address these issues.

### Public health value proposition for NGRVs

To better understand potential market success of NGRVs within the current rotavirus vaccine landscape, PATH developed a value proposition to understand the public health value of NGRVs to help inform decisions by international agencies, funders, vaccine manufacturers, and countries. The value proposition has three main components: (1) a mixed-method study to elicit both national stakeholder (NS) and healthcare provider (HP) views on the feasibility and acceptability (F&A) of real and hypothetical NGRV candidates with different characteristics and benefits (HPs views are the subject of this paper; NS findings are reported elsewhere [21]); (2) economic modeling to project the impact and cost-effectiveness of real and hypothetical NGRVs compared to currently available LORVs [22]; and (3) informed by findings from #1 and #2, demand forecasting to quantify potential market sizes for new vaccine options.

Crowded immunization schedules and consequent strain on the health system [23], coupled with concerns about vaccine hesitancy linked to vaccine characteristics [24], are increasingly important considerations for LMIC decision-makers when deliberating new vaccine introductions (NVIs) and product switches [25–27]. Though LMIC stakeholders are rarely consulted on vaccines in the development pipeline, their perspectives offer vital context for developing products that fit their operational realities and help meet child health goals. Within the broader value proposition, the F&A study was done to bring LMIC perspectives into the global dialogue on future NGRV research and development directions. HPs, who are most impacted by changes to the vaccine schedule, are best positioned to provide insights on what matters most from a delivery perspective.

To this end, this study addressed the questions: Would healthcare providers prefer to deliver new rotavirus vaccines compared to existing products? Why or why not?

## Materials and methods

### Sample

The five countries included in the study were purposively selected to represent different geographic regions and Gavi, the Vaccine Alliance co-financing and graduation status. This information, along with other country characteristics, can be found in Table 1. All five countries have introduced an LORV within the last 7–12 years.

**Table 1:** Study country characteristics.

Ref	WHO Region	AFRO				PAHO
	Country & Sample Size	Ghana (n = 10)	Kenya (n = 11)	Malawi (n = 14)	Senegal (n = 15)	Peru (n = 14)
**Economic Indicators**						
(1)	Country classification	LMIC	LMIC	LIC	LMIC	UMIC
(2)	Gross National Income ($US per capita 2019)	$2,220	$1,750	$380	$1,450	$6,740
**Diarrheal Disease and Rotavirus Disease Burden Indicators**						
(3)	U5 mortality from diarrheal disease (per 100,000), 2019	67.64	122.31	103.60	139.50	12.39
(4)	U5 mortality from rotavirus (per 100,000 children), 2019	16.42	74.94	22.21	63.46	2.08
**Vaccination Program and Gavi Indicators**						
(5)	Official/estimate % coverage (2019)					
	DTP3	97	91	95	95	88
(6)	Gavi co-financing status (2019)	Preparatory transition phase	Preparatory transition phase	Initial self-financing	Initial self-financing	NA
**Rotavirus Vaccine**						
(7)	Introduction date	Apr 2012	Jul 2014	Oct 2012	Nov 2014	Jan 2009
	RV vaccine	ROTARIX (until2019); ROTAVAC	ROTARIX	ROTARIX	ROTARIX	ROTARIX

(1) https://www.gavi.org/programmes-impact/country-hub; LMIC = Lower Middle Income Country ($1,036-$4,045 per capita); LIC = Lower Income Country (≤$1,035 per capita); UMIC = Upper Middle-Income Country ($4,046-$12,535 per capita).

(2) https://www.gavi.org/programmes-impact/country-hub.

(3–4): Global Burden of Disease Study 2019 (GBD 2019). Available from: http://ghdx.healthdata.org/gbd-results-tool.

(5) WHO Global Health Observatory: https://www.who.int/data/gho/data/indicators/indicator-details/GHO/diphtheria-tetanus-toxoid-and-pertussis-(dtp3)-immunization-coverage-among-1-year-olds-(-).

(6) https://www.gavi.org/sites/default/files/document/gavi-co-financing-policypdf.pdf.

(7) https://view-hub.org/.

In each country, three to five primary healthcare facilities located within a two-hour drive from the interviewer were included in the study. Facilities farther than 2 hours’ drive were excluded due to budget, time, and logistical constraints. With the assistance of facility in-charges, the lead investigator in each study country recruited one to five providers who currently administered childhood vaccinations to participate in individual interviews, with a goal to recruit 10–15 HPs per country. A total of 64 HPs participated in this study.

### Comparison vaccines

HP interviews focused on three comparison vaccines: a comparator LORV and two hypothetical NGRVs. The first NGRV is a standalone injectable vaccine, iNGRV, modeled after the trivalent P2-VP8 subunit vaccine candidate and presented as a three-dose schedule and administered via intramuscular injection [19]. The trivalent P2-VP8 candidate may eliminate intussusception risk because it is injected and may have the potential to be combined with existing DTP-containing combination vaccines. The second NGRV is an oral vaccine, oNGRV, modeled after the neonatal rotavirus vaccine candidate RV3-BB and delivered as an initial birth dose followed by two doses given in the routine infant schedule [18]. RV3-BB may reduce the risk of intussusception and provide infants early protection against rotavirus infection [18]. Both trivalent P2-VP8 and RV3-BB are in late-stage clinical development.

Country-specific visual aids containing known or assumed profile attributes of LORVs and NGRVs were displayed to the interviewee to facilitate question comprehension and interview flow. Without identifying vaccines by name, visual aids included information for each vaccine in the comparison on:

1. Presentation (e.g., 1 dose/plastic tube, liquid form)
2. Route of administration (oral vs. injectable)
3. Schedule and doses (age of each dose visit and number of doses)
4. Cold chain volume per fully immunized child (FIC)

To focus on the feasibility aspects of delivering NGRVs, efficacy assumptions were not provided to HPs. Table 2 details assumptions used to prepare the visual aids.

**Table 2:** Rotavirus vaccine product profiles.

	ROTARIX^1^	ROTAVAC^1^	ROTASIIL^1^	iNGRV^2^	oNGRV^3^
Presentation	Plastic strip of 5 tubes	5-dose vial	2-dose lyophilized vial plus diluent	2-dose vial without preservative	Plastic strip of 5 tubes
Route of administration & dosage	Oral; 1.5 mL	Oral; .5 mL (5 drops)	Oral; 2.5mL	Injectable; .5mL	Oral; 1mL
Schedule & doses	2 doses at 6 & 10 weeks	3 doses at 6, 10, 14 weeks			3 doses: neonatal, 6 and 10 weeks
Cold chain volume per fully immunized child (cm3)	23.6	12.6	31.6	46.2	23.6

^1^Gavi rotavirus vaccine profiles: https://www.gavi.org/sites/default/files/2021-11/Gavi-Rotavirus-vaccines-profiles-Nov-2021.pdf.

^2^A O’Neill, personal communication, June 12, 2019.

^3^Bines JE, At Thobari J, Satria CD, Handley A, Watts E, Cowley D, et al. (2018). Human Neonatal Rotavirus Vaccine (RV3-BB) to Target Rotavirus from Birth. The New England journal of medicine. 378:719–30.

### Interviews

One-on-one interviews lasting ≈40–45 minutes followed a structured interview guide comprised of fixed-choice and open-ended questions (S1 File). Interviewers strictly followed the guide. Data were collected on each HP’s experiences and roles, their involvement in NVIs, perceptions about the health impact of rotavirus and LORV introduction, and their preferences for different vaccine options. Vaccine preference questions proceeded in three steps, summarized in Table 3.

**Table 3:** Summary of vaccine comparisons & delivery scenarios.

**Step 1: Healthcare providers asked to assume that all vaccines in the comparisons:** • Have a shelf-life of 24 months at 2–8°C • Are comparable, with good safety profiles				
**Step 2: Healthcare providers indicate vaccine preference on four core comparisons (C1-C4)**				
First, healthcare providers select a comparator LORV, which will subsequently be used in C2-C4	C1	LORV 1	LORV 2	LORV 3
Key question 1: Would a standalone iNGRV be preferrable to oral vaccine options (LORV and oNGRV)? Why/why not?	C2	LORV		iNGRV
	C4	oNGRV		iNGRV
Key question 2: What are HP preferences for LORV compared to oNGRV, and what do HPs view as advantages, challenges, or concerns regarding delivery?	C3	LORV		oNGRV
**Step 3: Healthcare providers asked about delivering combination and co-administration scenarios**				
Key question 3: If iNGRV is included as part of existing penta/DTP-containing vaccine*, what concerns would health providers have, if any, about administering this combination product?				
Key question 4: If iNGRV is found to offer substantially higher protection if given alongside existing LORVs, could HPs feasibly co-administer both vaccines? What would be the challenges?				

*DTP = diphtheria, tetanus, pertussis; pentavalent = DTP plus hepatitis B and *Haemophilus influenzae* type b (Hib).

First, in comparison 1 (C1) HPs were asked their preference among three currently available LORV products. Then, based on their selection, the preferred LORV product was used for the remainder of the interview to compare against iNGRV and oNGRV candidates in comparisons C2 and C3. For C4, HPs were asked to compare the two new vaccine candidates: iNGRV and oNGRV. For each comparison, HPs were asked to indicate which vaccine they preferred and to explain why. Additional open-ended questions elicited providers’ thoughts on co-administered schedules where both LORV and iNGRV are given, as well as on concerns, if any, about the inclusion of iNGRV into the existing DTP-containing vaccine as a combination presentation.

### Data processing

iPads were used to record the interviews and collect data from fixed-choice questions using Research Electronic Data Capture (REDCap) tools [28]. Quantitative data captured through REDCap were automatically uploaded to datafiles. Audio-recordings were translated into English, if needed, and transcribed in full. Transcripts were coded using NVivo 12 Pro [29]. Where discrepancies between the transcript and quantitative data were found, the quantitative datafile was corrected to align with responses recorded in the transcript.

### Data analysis

Textual and quantitative data were analyzed independently and together applying cross-over mixed analytic approaches [30]. Frequency distributions on quantitative data were determined. Textual data were coded following an initial broad coding scheme that was iteratively refined through team-based, inductive coding [31, 32]. Select codes/categories were reduced to numeric variables [33] to reveal thematic patterns, discern within-category diversity, and to merge with quantitative data for cross-over analysis. Consensus-based coding/categorizing of data was done by two primary coders (JM and JP), with intermittent verification by all study investigators.

#### Preference drivers

On each comparison, replies to the question, *Why did you select vaccine X*? were coded to identify preference drivers. Driver codes were grouped into successively broader categories, eventually assigning all codes to one or more main drivers. FEASIBILITY includes references to programmatic and operational issues to store, transport, and administer the vaccine according to the schedule shown in the visual aid; ACCEPTABILITY includes references to potential child caregiver hesitancy to injections and HP resistance to administering injections, as well as positive and negative sentiments expressed for oral vaccines; SAFETY includes references to injection safety, concerns about administering vaccines to neonates, AEFIs, and issues with oral administration and vomiting/aspirating; PUBLIC HEALTH IMPACT includes references to children possibly not receiving full doses of oral vaccines due to spitting up and vomiting, perceived coverage concerns, and perceptions regarding efficacy of oral versus injectable vaccines. Coded replies concerning NVI challenges and opportunities based on HP experiences were grouped into three main categories: Community Acceptability; Training and Knowledge Needs; and Immunization Operations and Logistics. These are discussed in further detail in the Results and Discussion sections.

### Ethics

Ethical approval to conduct this study was obtained from PATH’s Research Ethics Committee, the Ghana Health Service Ethics Review Committee, the Kenyatta National Hospital-University of Nairobi Research Ethics Committee, Malawi’s National Health Sciences Research Committee, Peru’s Via Libre Comité lnstitucional de Bioética, and Senegal’s Comité National d’Ethique pour Ia Recherche en Santé. Written informed consent was obtained prior to conducting interviews.

## Results

### NVI experiences

All 64 HPs were from urban and peri-urban primary health facilities providing routine vaccination via fixed and outreach services All HPs were certified community/public health nurses who routinely administer vaccinations, most of whom were well-experienced (Table 4). Certifications varied slightly by country context. 46 of the 64 providers interviewed said they had been involved in an NVI, and 38 reported previous experience with vaccine switches. The need for comprehensive HP training and early community sensitization were commonly cited as facilitators for successful NVIs.

**Table 4:** Healthcare providers by country and years of experience in vaccine administration.

Country	Total sample	<1 year	1 to <5 years	5 to <10 years	10 to <20 years	20 + years
Ghana	10*	-	1	6	2	-
Kenya	10	-	-	5	3	2
Malawi	14	2	1	1	6	4
Peru	15	1	2	6	5	1
Senegal	15	-	4	4	5	2
**TOTAL**	**64**	**3**	**8**	**22**	**21**	**9**

*Information from one Ghana interview not captured here due to audio issues.

### Rotavirus and LORV impact perceptions

36 (56%) of HPs interviewed considered rotavirus to be a “very serious problem and one of the leading causes of child deaths” in their respective countries, while 21 (33%) considered rotavirus to be “a serious problem, but not among the top causes of child deaths”; only 5 considered rotavirus “not a serious problem compared to other childhood diseases”, and 2 “didn’t know.” In terms of the impact of LORVs, 22 (34%) HPs agreed with the statement that LORVS have “significantly reduced under-five mortality,” 39 (61%) agreed that they have “helped but more is needed”; none agreed that LORVs “have not led to substantial changes in childhood diarrheal deaths,” and 3 “did not know.” Distribution of these findings was very similar across study countries despite differences in rotavirus under-five mortality rates and vaccine coverage (Table 1).

### Vaccine preferences

**Fig 1:**
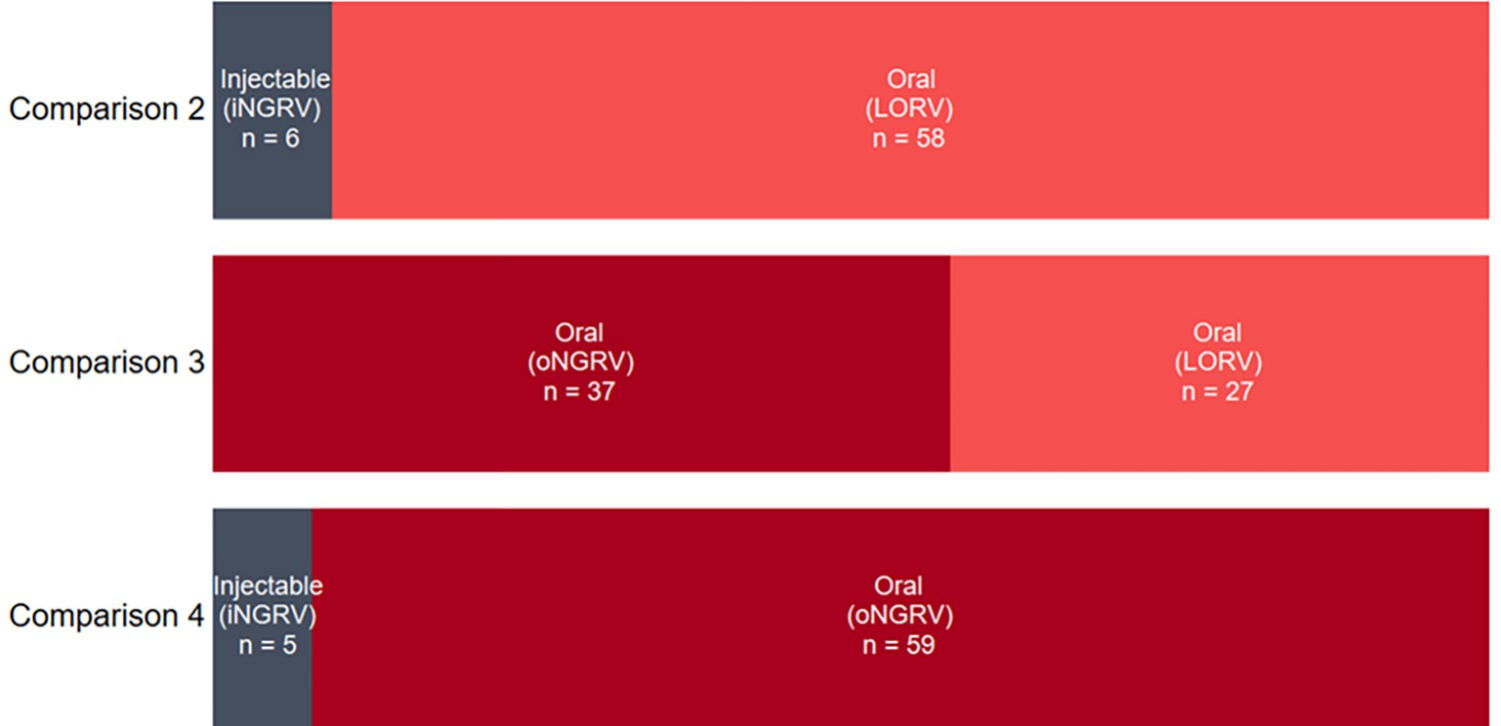
Healthcare provider preferences among vaccine comparisons (C2-C4).

**Fig 2:**
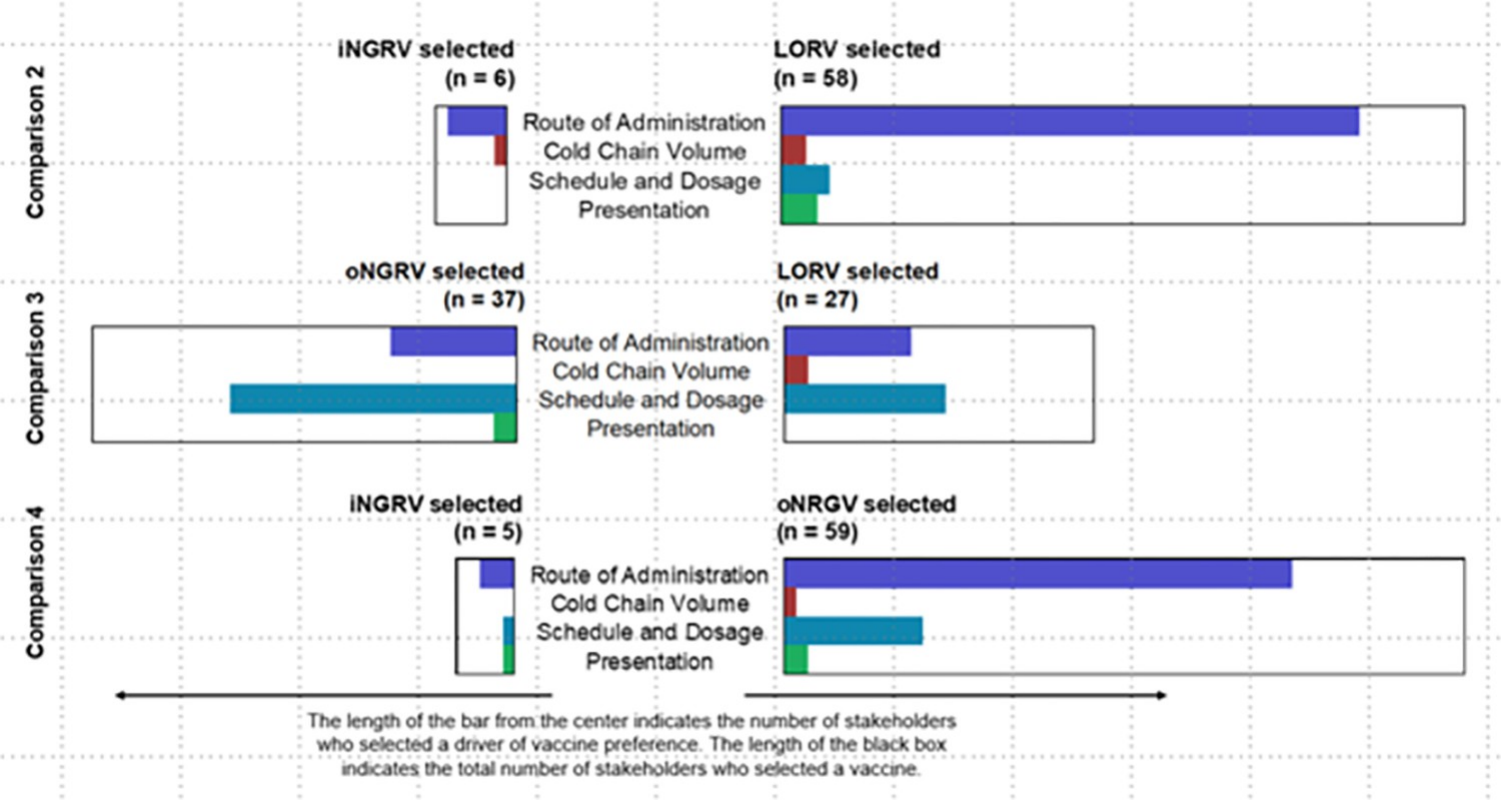
Vaccine preferences by main attribute selected.

As summarized in Fig 1, HPs strongly preferred oral vaccine options over iNGRV (Comparisons 2 and 4). HP preferences were mixed when comparing LORV versus an oNGRV requiring a birth dose (Comparison 3), with a modest majority preferring the neonatal option over LORV. Fig 2 shows preferences drivers derived from coded and categorized qualitative data. These findings are detailed below.

#### Key question 1: Would a standalone iNGRV be preferrable to oral vaccine options (LORV and oNGRV)? Why or why not?

As illustrated in Fig 1, 58 (91%) of providers preferred LORV over iNGRV, and 59 (92%) preferred oNGRV to iNGRV, with the route of administration cited as the main reason for the preferences in both comparisons. In contrast to frequent descriptions of oral dose delivery as “easier,” “faster,” “more acceptable,” and “safer,” a robust majority of the HPs indicated one or more concerns related to delivery by injection. The “overwhelming” number of injectable vaccines in the schedule and the number of injections given in a single visit were, together, the most frequently cited reason for preferring LORV (n = 49) and oNGRV (n = 44). Many HPs reported that mothers in their countries strongly dislike injections. Additionally, many expressed their own distress at inflicting pain when giving injections to children and the challenge of managing caregiver resistance. Examples from Ghana and Senegal:

“There are already three injectables. This one would make four. The mothers complain about the third one, which is at 14 weeks. They know [it’s coming], but the moment you tell them… they shout… Even you, as a health worker, injecting first, second, third, come to the fourth one, I believe sometimes you don’t feel okay….” (G_007)

“The feedback we receive from the mothers is ‘Can you stop?’. There are too many injections. This is what the mothers tell us, repeatedly.” (S_006)

Some HPs reported that too many injections are given, particularly on the 6, 10, and 14 week schedule (n = 12), which is the schedule for iNGRV. According to one HP in Ghana, this can also cause potential vaccine administration errors:

“If you are not careful, you think you’re giving a penta, or you’ve given a pneumococcal, whilst you have not… there will be confusion if there are too many.” (G_001)

With respect to oNGRV, HPs were supportive of children getting earlier protection against rotavirus via a neonatal dose, with the schedule and doses cited as the second most common preference driver after route of administration (12/59).

Of the few providers who preferred the iNGRV product in these comparisons (n = 5 when compared to LORV; n = 6 compared to oNGRV), selected iNGRV in both comparisons. These HPs reported that an injectable vaccine would eliminate the risk of the child “spitting up”, “choking” or “vomiting”, which ensures the complete volume of dose is given and the child is fully vaccinated and protected against rotavirus. One individual perceived safety risk and possible contamination with administering oral vaccines. Interestingly, even for HPs who preferred the oral products, spitting up, choking, and vomiting were all cited as challenges with oral administration (n = 13).

#### Key question 2: What are HP preferences for LORV compared to oNGRV, and what do they view as advantages, challenges, or concerns regarding delivery?

As Fig 1 shows, providers were more evenly split in preferences for oNGRV or LORV, though a majority favored the new neonatal option (n = 37) compared to LORV (n = 27); Fig 2 shows the vaccine attributes that most influenced their selections. While HPs in both groups had similar concerns, reflected in the Driver Category column of Table 5, specific perceptions and issues raised by the two groups were notably different and sometimes contrasting.

**Table 5:** Main preference drivers for oNGRV and LORV*.

Driver Category	oNGRV Selected (n = 37)	LORV Selected (n = 27)
**Epidemiology (protection)**	N = 24, diarrhea prevalent in newborns; infants unprotected; poor hygiene (“babies passed around by family”)	N = 5, diarrhea a problem in older children, not newborns; neonatal protection not needed; vaccine won’t help
**Safety and efficacy**	N = 4, smaller dose (1 mL) safer; neonate immune system needs boost	N = 10, larger dose (1.5 mL) more effective; neonate immune system too young for vaccination—won’t respond; could be overwhelmed by another vaccine given in this neonate visit
**Coverage**	N = 5, assure child receives at least one dose; mothers more accepting right after birth	N = 3, midwives forget to administer or record neonatal vaccinations; neonatal dose misses home and weekend births
**Organization, capacity, schedule**	N = 10, delivering neonatal vaccines already; could help alleviate heavy burden in routine EPI services	N = 16, LORV known; no change or training needed; avoids MNCH-EPI integration challenges with neonatal dose

*Individuals may have more than one preference driver.

For those who selected oNGRV, the main driver was schedule and doses (25/37), with many providers pointing out that since neonatal OPV and BCG doses are already given at birth, adding one more oral vaccine would not be a problem. When asked about possible challenges in providing a neonatal rotavirus vaccine, almost half of all HPs described one or more challenges, including 10 individuals who selected oNGRV over LORV. In total, 42 neonatal challenges were described by 33 HPs representing all five countries in the sample (Table 6). Issues related to maternal and child health and EPI system integration was the most prominent challenge cited (n = 15), mainly framed as midwives “forgetting” to administer or document neonatal vaccines, so HPs “don’t know if the child received the vaccine or not.” Some HPs had safety concerns and/or were skeptical about neonates being able to handle a birth dose due to “immature immune systems” or that another neonatal vaccine given alongside OPV and BCG would be “too much” for a newborn.

**Table 6:** Healthcare provider neonatal dose concerns by type.

Neonatal delivery challenges cited* (n = 33)	
Integration with maternal, newborn and child health (MNCH; midwives forget to deliver and/or record)	**15**
NVI/switch challenges, generally; educating mothers, specifically	**8**
Neonate immature (dose too strong, can’t ingest, immune system won’t respond)	**7**
Coverage (home births, weekend births)	**6**
Safety concerns (negative reactions, too much with other neonatal antigens)	**4**
Other, unclear	**3**

*Individuals may have more than one concern.

Additionally, a few HPs mentioned a distinct issue with current LORV delivery, mainly that caregivers assist with giving doses and/or positioning the baby for ease of administration. It merits further investigation as to how often caregiver assistance occurs and if its possible caregivers are not just assisting but are administering vaccine, and if this is fully supervised by an HP as this may have implications as to whether children are getting fully immunized. Spitting up, choking, and vomiting were all cited as issues with administration (n = 13), even among HPs who preferred oral products.

#### Key question 3: If iNGRV is included as part of existing DTP-containing vaccine, what concerns would HPs have, if any, about administering this combination product?

HPs were asked to indicate any concerns about giving iNGRV as part of the existing DTP-containing vaccine. The majority stated they had no concerns (46/64), and even expressed enthusiasm, as it would free up cold chain storage currently reserved for LORV as well as eliminate LORV in the visit schedule. HPs reported it would “make the job easier” and “kill two birds with one stone.” However, 18/64 providers voiced concerns about including iNGRV in the combination vaccine. The HPs reported that “mothers already complain” about the DTP-pentavalent vaccine because it is “very painful”, and that DTP has a “very strong reaction”. They worried that adding iNGRV would be “too strong” and might exacerbate these issues. Those with concerns about an iNGRV-DTP combination commonly expressed multiple issues (Table 7).

**Table 7:** Healthcare provider concerns for including iNGRV in a DTP-containing combination vaccine by type.

iNGRV-DTP-containing combination vaccine challenges cited* **(n = 32)**	
Caregiver acceptability (already concerned/complain about side effects of penta; used to LORV so why change?)	**12**
Safety concerns (increased pain, fever, other AEFIs)	**11**
Potential inferiority reactions with other DTP-containing antigens	**5**
Other, unclear	**4**

*Individuals may have more than one concern.

#### Key question 4: If iNGRV is found to offer substantially higher protection if given alongside existing LORVs, could HPs feasibly co-administer both vaccines? What would be the challenges?

To explore the feasibility of delivering both iNGRV and LORV, HPs were presented with the schedule of co-administering both iNGRV and LORV at 6 and 10 weeks, and a third dose of iNGRV at 14 weeks. HPs were then asked if they could deliver both vaccines and if they anticipated any concerns or challenges. Table 8 shows the breakdown of these results by country, with 30/64 HPs reporting they could feasibly deliver both vaccines according to the co-administration schedule, 27/64 could not, and 7/64 expressed uncertainty or needed caveats, like clear, convincing messaging for caregivers explaining the rationale for giving both vaccines. Overall, HPs in Malawi and Peru were more amenable to this delivery schedule, while HPs in Kenya and Senegal were more reluctant to this schedule. Providers who supported the co-administration schedule reported that if the assumption around increased public health benefit were proven, the complex delivery and potential difficulty in sensitizing caregivers would be “worth it.”

**Table 8:** Healthcare provider responses for delivering an LORV + iNGRV co-administration schedule.

Country	Could give co-admin schedule	Could not give	Unsure/Maybe with caveats
Ghana	5	5	-
Kenya	2	6	2
Malawi	10	3	1
Peru	8	4	3
Senegal	5	9	1
**TOTAL (n = 64)**	**30**	**27**	**7**

HPs reluctant to support the co-administration schedule cited concerns with community acceptability, stating they could give both vaccines, but the “problem” would be with caregivers. These providers were not convinced caregivers would “understand” giving the “same” vaccine:

“The mother is going to say, ‘Why are you using [iNGRV] if you are already administering [LORV]?’ It tends to generate doubt in the population… and in health we can’t give ourselves the luxury of hesitating because it really generates rapid alarm among the population. Therefore, we should adopt only one method, either the oral or the injectable.” (P_007)

Many providers struggled with the logic of giving two different vaccines for the same disease instead of manufacturers pursuing a more effective single-dose vaccine:

“Why can’t it be that for that higher protection, you just make one…I don’t understand. It can just be the one formulation giving that higher protection.” (K_006)

## Discussion and conclusion

Findings from this study add to the existing literature on vaccine assessments, value propositions, and investment cases [34–37], including a complementary paper reporting on national stakeholder findings regarding NGRVs [21]. Interviews with HPs on vaccine preferences elucidated potential barriers and facilitators for NGRV delivery, which align with previously published findings around vaccine feasibility and acceptability in LMIC settings [23, 38]. While it was unsurprising that providers are overwhelmingly reluctant to administer another injection to children as part of EPI programming, it was interesting to find that. oral vaccine options also presented challenges with administration, including vomiting, choking, and spitting up part of the dose. However, continued HP preference for oral options suggests these challenges are not strong enough to overcome the resistance to injectable products, especially if an oral alternative is already available.

When comparing oral vaccines, HPs who selected LORV expressed very similar reasons for their preference as HPs who selected oNGRV, perhaps illustrating differences and nuances among country EPI programs. Additionally, the perceived weakness of maternal and child health integration with EPI programming and concerns over midwives administering and documenting the oNGRV neonatal dose merits further investigation, as it has broader implications for other neonatal vaccines.

Though HPs were divided over a co-administration schedule (iNGRV with LORV) to improve protection against rotavirus, those who were supportive of co-administration cited the increased public health impact as motivation. Given the HP openness in potentially delivering this co-administration schedule as well as the concerns over community acceptability suggests a need to better understand caregiver perspectives about co-administration of multiple vaccines for the same antigen, possibly drawing on OPV/IPV experiences.

The possibility of adding iNGRV to the existing DTP-containing vaccine was very appealing to the majority of providers, as it would streamline EPI schedules and eliminate the administration of LORV. However, a few expressed the perception that DTP is already painful for the child and AEFIs are common, so wondered if adding iNGRV would worsen these issues. Concerns over adding an antigen to DTP warrants further understanding and development of advocacy and communication messaging.

### Study limitations

Restricting the inclusion criteria to those facilities within two hours of the study management office means rural HP perspectives are not represented. Unlike with the national stakeholder interviews, information on vaccine efficacy and public health impact were not included attributes in the vaccine product criteria presented to HPs for the comparisons, since we mainly wanted to focus on feasibility of delivery. Therefore, it is unknown the degree to which this information would influence HP preference for a higher efficacy iNGRV standalone product despite strong reluctance to administer another injectable vaccine. However, given HP preferences for oNGRV due to earlier protection for the child, as well as supportive responses for the co-administration scenario which assumed that adding iNGRV would increase efficacy, it seems having this knowledge might sway some opinions about product preference and willingness to advocate to caregivers.

### Implications

In order to translate this feedback into recommendations for improving NVI processes, country investigators took specific challenges mentioned within these emerging themes and developed actionable interventions to address these issues. Below are a few critical points which merit further consideration and engagement as a result of this study.

HP Engagement in NVIs: HPs are critical gatekeepers for vaccine acceptability and uptake in their respective communities. However, providers are frequently left out of important discussions that influence national vaccine policymaking and are rarely consulted when it comes to upstream development of new vaccine products. Having more opportunities for providers to share insights, experiences, and represent the voice of the communities they serve might help to better inform policymaking as well as the design of products in the vaccine development pipeline, which could ultimately increase vaccine uptake.

Vaccine Training & Knowledge Needs: HPs requested more information on infectious disease epidemiology as well as vaccine safety and efficacy. Information on the rationale and advantages of vaccine presentation (i.e., why so many injectable vs. oral products), dosage, and scheduling would better equip HPs with the ability to respond to caregiver questions and concerns.

HP Support for Injection Fatigue and Vaccine Confidence: Providers interviewed for this study frequently brought up the issue of injection fatigue, both in terms of administering injectable vaccines to children and also caregiver willingness to having their child vaccinated. Recent studies show a global decline in vaccine confidence among HPs, which could potentially jeopardize vaccine uptake if their willingness and ability to act as advocates or recommend vaccination to caregivers is diminished [24, 39–41]. HPs share similar safety concerns as parents and are influenced by the same myths, rumors, and social media posts, the circulation of which can be overwhelming.

While access to clearly explained scientific information is important, other mitigation strategies are also needed to address the social influence of anti-vaccine groups and vaccine hesitancy. While most NVI training efforts focus on HP community sensitization with caregivers, perhaps more HP-specific interventions targeting trust and confidence-building around vaccines are needed.

Furthermore, due to time constraints and workload, HPs may not feel equipped to address vaccine hesitancy among parents nor know how to engage in a potentially sensitive topic. Though research focusing on VH among HPs has gained traction in recent years, more feedback from LMIC providers is needed to better understand the contexts for hesitancy among those delivering routine vaccinations, especially given the rapidly evolving childhood immunization landscape.

#### Organizational support

Advancements in vaccine development combined with the availability of Gavi co-financing support has had a powerful impact on access to lifesaving vaccines in LMIC settings, particularly over the last two decades. The number of vaccines offered through childhood immunization programs has increased, in some cases even doubled or tripled, in a relatively short period of time. While this progress is commendable, further inquiry into how this has impacted HP workload and morale, as well as health system capacity issues, must be examined to ensure sustainability, especially since “system failure” issues such as vaccine stockouts and service limitations due to staffing shortages can further erode community confidence in vaccines [41].

Soliciting feedback from HPs on their rotavirus vaccine product preferences and NVI experiences more generally resulted in insightful contributions to help inform the public health value proposition for NGRVs and beyond. The findings from HPs in this paper offer a complementary perspective to that of national stakeholders who were also interviewed as part of this study (Price et al). Additionally, an accompanying cost-effectiveness analysis by Debellut et al provides further economic rationale for NGRVs, in particular the iNGRV-DTP combination vaccine so favored by HPs as reported in this paper.

## Supporting information

S1 File HP interview guide. (DOCX)

S2 File Inclusivity in research. (DOCX)
