# Supplementary material for: Healthcare provider perspectives on delivering next generation rotavirus vaccines in five low-to-middle-income countries
Source: PLoS One. 2022 Jun 23;17(6):e0270369. doi: 10.1371/journal.pone.0270369 (PMC9223340; doi:10.1371/journal.pone.0270369)
Supplement: S1 File — (DOCX) [file pone.0270369.s001.docx]

**NGRV F&A Study**

**Health Provider Interview Guide**

**Version: 12 November 2019**

X.0 HP Unique ID: ___ - ___ ___ - ___ ___ ___

X.1 Have you turned on the audio recording?

⃝ Yes

⃝ No

X.2 Speak into the recording:

“Starting the interview with respondent [HP Unique ID]

X.3 Date interview was conducted: DD/MM/YYYY

| **Country:** | 1 | Ghana | 5 | Peru |
| --- | --- | --- | --- | --- |
|  | 2 | Kenya | 6 | Senegal |
|  | 3 | India |  |  |
|  | 4 | Malawi |  |  |

**Introduction & Interview Initiation**

Thank you for agreeing to take part in this study. My name is………………..?

The intent of this interview is to understand what you, as health provider who administers vaccines, prefers most between vaccine options. As there are several new rotavirus vaccines currently being studied, the information we learn from you and other providers will be used to inform which of these new vaccine options should be pursued.

Please keep this in mind during the interview. I’m going to start by asking you a few background questions and then will ask you a series of questions asking you to compare different rotavirus vaccine product options.

**Section 1: Provider & Facility Profile**

1.1 Please start by telling me a little bit about yourself and your role in this facility.

*Do not need to type the answer – audio recording will capture*

1.2 Please tell which days of the week are vaccination services offered by this facility, whether facility- or community-based?

*Do not need to type the answer – audio recording will capture*

1.3 Does this facility provide vaccinations through community-based outreach programs?

*If yes, can you describe your role in providing vaccinations in outreach settings?*

*Do not need to type the answer – audio recording will capture*

1.4 About how many years have you been giving vaccinations?

*Do not need to type the answer – audio recording will capture*

1.5 What is your highest degree/qualification earned?

*Do not need to type the answer – audio recording will capture*

1.6 What is the most recent vaccination training you received?

*Do not need to type the answer – audio recording will capture*

1.7 Have you ever been part of a process when a new vaccine is introduced?

*If yes, ask:* Tell me about that process.

*Probe:* What was your role? What went well? What was challenging?

*Do not need to type the answer – audio recording will capture*

Thank you very much for those replies. I’d now like to turn the focus of my questions on rotavirus.

1.8 From your perspective, which statement best describes the seriousness of rotavirus public health problem in [COUNTRY]?

⃝ A very serious problem and one of the leading causes of child deaths

⃝ A serious problem, but not among the top causes of child deaths

⃝ Not a very serious problem compared to other childhood diseases

⃝ Don’t know

1.8.1 What makes you respond this way?

*Do not need to type the answer – audio recording will capture*

1.9 Do you provide rotavirus vaccination at your facility?

*Do not need to type the answer – audio recording will capture*

1.10 From your perspective, which statement best describes the impact that rotavirus vaccine provision has on diarrheal disease in [COUNTRY]?

⃝ Has significantly reduced under-five mortality

⃝ Has helped to reduce under-five deaths, but more needs to be done

⃝ Has not led to substantive changes in childhood diarrheal deaths

⃝ Don’t know

1.10.1 What makes you respond this way?

*Do not need to type the answer – audio recording will capture*

1.11 I have one final question before we move on to the vaccine comparisons. How would you describe how a particular vaccine’s efficacy affects you when giving them to children? *Possible probes*: *If it does affect them in any way*, Can you give me a specific example? *Then probe further as needed*.

*Do not need to type the answer – audio recording will capture*

**Section 2: Vaccine Comparisons**

Thank you for your replies so far. I now want to transition to questions that will ask you to compare different vaccine options. There will be four comparisons in all. For each comparison, I’m going to show you information about each vaccine being compared and then I’ll ask you to tell me which of them you would prefer and why. Please turn to page 2. This is an explanation of each of the four vaccine attributes I will ask you to compare between the different vaccine comparisons.

Please turn to page 3. Here are the two assumptions about all the vaccines I will ask you to compare. All vaccines in these comparisons are equally safe and effective. We also assume all vaccines in these comparisons will have a shelf-life of 24 months at 2-8°C.

Do you have any questions about these assumptions?

Okay, thank you. Let’s get started with the first vaccine comparison.

**Visual Aid: Comparison 1**

Please flip to page 4. You should be looking at Visual Aid: Comparison 1 in blue, which is showing information for three existing rotavirus vaccines. Take a moment to review the information shown in the chart and let me know when you are ready or if you have any questions.

|  | Existing Vaccine 1 | Existing Vaccine 2 | Existing Vaccine 3 |
| --- | --- | --- | --- |
| Please select which vaccine is your first choice |  |  |  |
| Please select which vaccine is your second choice |  |  |  |
| (do not ask respondent) select remaining vaccine as third choice |  |  |  |

*Allow the participant time to review the information and keep it displayed throughout the question series.*

2.1 Why did you select XXX as your first choice?

*Do not need to type the answer – audio recording will capture*

2.1.1 Even though you selected XXX as your first choice, which attribute did you find least important in this decision? **[pause]** Why did you select this attribute?

⃝ Presentation

⃝ Route of administration

⃝ Schedule & dosage

⃝ Cold chain volume

Now please turn to page [X], you should be looking at Visual Aid: Comparison 2 in [color]. This chart compares [selected existing vaccine] with a new rotavirus vaccine that is currently being studied and may become available by 2025.

Please take a moment to review and compare the information in the chart, as the injectable vaccine differs from the oral vaccine in several other aspects. Let me know when you are ready or if you have any questions.

**Visual Aid: Comparison 2**

2.2 Now please select which of the two vaccines you prefer.

⃝ New vaccine

⃝ Existing vaccine

2.2.1 Tell me why you have selected the vaccine you did.

*Do not need to type the answer – audio recording will capture*

2.2.2 Which statement best describes the strength of your preference for [PREFERRED VACCINE]?

⃝ Very strong

⃝ Moderately strong

⃝ I don’t have a strong preference

2.2.3 Looking at all the attributes shown, which one did you find most important or influential to your decision and why?

⃝ Presentation

⃝ Route of administration & dosage

⃝ Schedule

⃝ Cold chain volume

2.2.4 Of these same attributes, which one would you describe as least important or influential to your decision and why?

⃝ Presentation

⃝ Route of administration & dosage

⃝ Schedule

⃝ Cold chain volume

Now please turn to page [X], you should see Visual Aid: Comparison 3 in [color]. This comparison is between a new oral neonatal rotavirus vaccine (meaning it is given shortly after birth, with BCG and other vaccines given during this time) and your preferred existing vaccine from the previous comparison. Please review the information and let me know when you are ready to continue.

**Visual Aid: Comparison 3**

2.3 As you did earlier, please select which of the two vaccines you would prefer.

⃝ New neonatal vaccine

⃝ Existing vaccine

2.3.1 Tell me why you have selected the vaccine you did.

*Do not need to type the answer – audio recording will capture*

2.3.2 Which statement best describes the strength of your preference for [PREFERRED VACCINE] over [LESS PREFERRED VACCINE]?

⃝ Very strong

⃝ Moderately strong

⃝ I don’t have a strong preference

2.3.3 Looking at all the attributes shown, which one did you find most important or influential to your decision and why?

⃝ Presentation

⃝ Route of administration & dosage

⃝ Schedule

⃝ Cold chain volume

2.3.4 Of these same attributes, which one would you describe as least important or influential to your decision and why?

⃝ Presentation

⃝ Route of administration & dosage

⃝ Schedule

⃝ Cold chain volume

2.4 Thinking about the neonatal option, tell me what you think would be the major challenges to deliver this vaccine? How so? /Why?

2.4.1 What practical steps could be taken to overcome these delivery challenges?

**Visual Aid: Comparison 4**

Now please turn to page [X], you should see Visual Aid: Comparison 4 in orange. This comparison is between the new oral neonatal rotavirus vaccine from the previous comparison and the new injectable vaccine from the earlier comparison. Please review the information and let me know when you are ready to continue.

2.5 As you did earlier, please select which of the two vaccines you would prefer.

⃝ New neonatal vaccine

⃝ New injectable vaccine

2.5.1 Tell me why you have selected the vaccine you did.

*Do not need to type the answer – audio recording will capture*

2.5.2 Which statement best describes the strength of your preference for [PREFERRED VACCINE] over [LESS PREFERRED VACCINE]?

⃝ Very strong

⃝ Moderately strong

⃝ I don’t have a strong preference

2.5.3 Looking at all of the attributes shown, which one did you find most important or influential to your decision and why?

⃝ Presentation

⃝ Route of administration & dosage

⃝ Schedule

⃝ Cold chain volume

2.5.4 Of these same attributes, which one would you describe as least important or influential to your decision and why?

⃝ Presentation

⃝ Route of administration & dosage

⃝ Schedule

⃝ Cold chain volume

**Section 3: Co-Admin & Combo Options**

3.1 There is the possibility of the new injectable vaccine being included as part of the existing penta/DTP vaccine. What concerns would you have, if any, about giving a new rotavirus vaccine as part of the existing penta/DTP?

3.2 Finally, there is also the possibility that giving both a new injectable vaccine with the existing live oral rotavirus vaccine would provide substantially higher protection against rotavirus. Please turn to the next page. You should see Visual Aid 5. Thinking about giving both a new injectable *and* oral doses, please tell me if you think you could give these vaccines according to this schedule. What would be any concerns or challenges?

**Section 4: Interview Closure**

4.1 Other than the topics already covered in this interview, what else would say is important to country decision makers when they are deciding whether to introduce a new vaccine or switch vaccine products?

*Close the interview.*
